# Text-Book of Pharmacology, Therapeutics and Materia Medica

**Published:** 1885-12

**Authors:** 


A TEXT-BOOK OF PHARMACOLOGY, THERAPEUTICS AND MATERIA MEDICA. *By* T. LOUDER BRUNTON, M. D., D. SC., F. R. S. *Philadelphia:* LEA BROS & CO. *Chicago:* JANSEN, MCCLURG & CO.

This work has been expected for some years, and in explaining the long delay, the author refers to the time spent in verifying numerous statements collected from various sources.

It is divided into several sections, treating of general pharmacology, general pharmacy, inorganic materia medica, organic materia medica, vegetable materia medica and the animal kingdom.

Each section is properly subdivided, following excellent systems, and pains have been taken to include matter of real interest and importance for those who will use it. On the whole, it seems to be one of the fullest and best works on the subject in the language. J. H. L.

